# Retinal vein changes after treatment with aflibercept and PRP in high-risk proliferative diabetic retinopathy

**DOI:** 10.3389/fmed.2023.1090964

**Published:** 2023-03-09

**Authors:** Hui Zhao, Jundong Wang, Shuting Li, Ying Bao, Xiaoxia Zheng, Yuan Tao, Hong Wang

**Affiliations:** ^1^Department of Ophthalmology, Qilu Hospital of Shandong University, Jinan, China; ^2^Department of Ophthalmology, Fei County People’s Hospital of Shandong, Linyi, Shandong, China; ^3^Fourth People's Hospital of Jinan, Jinan, China; ^4^The Teaching Hospital of Shandong First Medical University, Jinan, China; ^5^Department of Ophthalmology, The Second People’s Hospital of Jinan, Jinan, China

**Keywords:** high-risk proliferative diabetic retinopathy, venous beading, retinal vein diameter, aflibercept, panretinal photocoagulation

## Abstract

**Objective:**

The objective of the study was to investigate the effectiveness of aflibercept and panretinal photocoagulation (PRP) in the treatment of proliferative diabetic retinopathy (PDR).

**Methods:**

A retrospective analysis was performed on 59 patients (59 eyes) with high-risk PDR who were treated with aflibercept and PRP between January 2018 and December 2019. The best corrected visual acuity (BCVA), central foveal thickness (CFT), and retinal vein diameter post-treatment were compared to those before the treatment.

**Results:**

The best corrected visual acuity (BCVA) at 6 months (0.49 ± 0.14 logMAR), 12 months (0.54 ± 0.15 logMAR), 18 months (0.48 ± 0.15 logMAR), and 24 months (0.51 ± 0.15 logMAR) post-treatment were superior to the pre-treatment measurement (0.65 ± 0.18 logMAR). The central foveal thickness (CFT) at 6 months (310.67 ± 52.53 μm), 12 months (295.98 ± 45.65 μm), 18 months (282.56 ± 43.57 μm), and 24 months (281.53 ± 51.16 μm) post-treatment were lower than the pre-treatment measurement (456.53 ± 51.49 μm); the retinal vein diameter at 12 months (310.13 ± 24.60 μm), 18 months (309.50 ± 31.58 μm), and 24 months (317.00 ± 27.54 μm) post-treatment were lower than the pre-treatment measurement (361.81 ± 30.26 μm).

**Conclusion:**

Aflibercept intravitreal injection and panretinal photocoagulation may morphologically reverse retinal vein diameter and venous beading in high-risk proliferative diabetic retinopathy.

## 1. Introduction

Diabetic retinopathy (DR) is a complication of diabetes and manifested as retinal microangiopathy. It occurs in many diabetic patients 5 to 10 years after the onset of the condition (1–3). As the most common complication of diabetes (4), diabetic retinopathy (DR) can lead to preventable blindness in working-aged adults (2, 3). However, many patients are not promptly diagnosed or treated until the development of high-risk proliferative DR. The high-risk proliferative DR is the late stage of DR progression and has been associated with poor outcomes and blindness (5).

Diagnostic criteria for high-risk proliferative DR were 1. Optic disk neovascularization of ≥1/4 to 1/3 of the optic disk diameter, with or without preretinal hemorrhage or vitreous hemorrhage; 2. Preretinal hemorrhage or vitreous hemorrhage with optic disk neovascularization or retinal neovascularization of ≥1/4 to 1/3 of the optic disk diameter. The diabetic retinopathy study recommended immediate panretinal photocoagulation (PRP) for eyes with high-risk PDR since the risk of severe vision loss in this population within 5 years was greater than 50% if the condition was untreated (6). In the past decade, anti-vascular endothelial growth factor (VEGF) agents were primarily used for the treatment of diabetic macular edema (7). The Diabetic Retinopathy Clinical Research (DRCR) Network Protocol S aimed to evaluate the effectiveness of ranibizumab compared to PRP in eyes with PDR. In this study, patients were randomized to ranibizumab 0.5 mg intravitreal injection monthly for 3 months. Any patient who developed progressive retinopathy despite monthly injections was allowed to receive PRP. At 2 years, ranibizumab provided better visual acuity outcomes, less visual field loss, fewer vitrectomies were required, and less development of center-involved DME when compared with the PRP group. The advantages of PRP were fewer visits, fewer injections, and greater cost-effectiveness in eyes without DME initially. Figueira et al. demonstrated that intravitreal injection of anti-VEGF agents was safe and was considered an option for high-risk PDR eyes in a study with a follow-up of 1 year. The outcome from intravitreal injection monotherapy or combination therapy was comparable or superior to that from PRP (8). The combination treatment of PRP plus an anti-VEGF drug may be the treatment of choice for PDR (9).

Previous retrospective studies elucidated peripheral reperfusion in ischemic areas of the retina in patients receiving anti-VEGF intravitreal injections, suggesting the potential of anti-VEGF therapies in reversing DR (10). This study aimed to determine the effectiveness of aflibercept combined panretinal photocoagulation in alleviating high-risk proliferative diabetic retinopathy, specific to retinal venous beading (VB), retinal vein diameter, best corrected visual acuity (BCVA), and central macular thickness (CFT).

## 2. Materials and methods

### 2.1. General information

**Figure 1:**
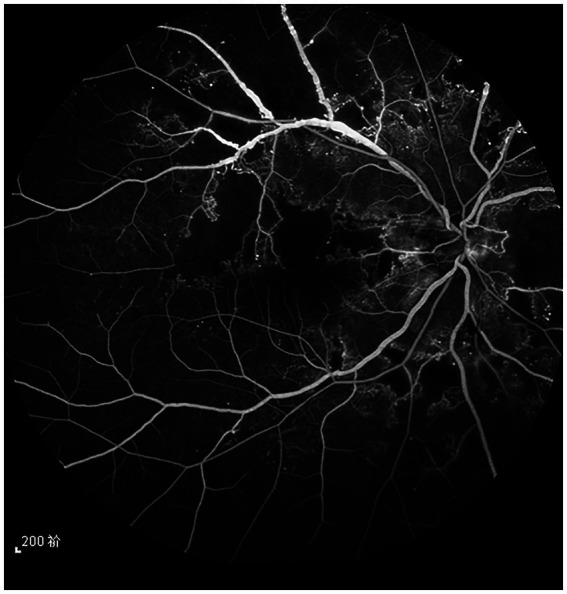
The fundus angiography image of the patient’s right eye before treatment. It can be seen that the retinal vein of the superior temporal branch is dilated, and the vein beading is obvious.

The data were collected from patients who were diagnosed with high-risk proliferative DR by fundoscopy, fundus fluorescein angiography (FFA), and optical coherence tomography (OCT) between January 2018 and December 2019 (Table 1). Diagnostic criteria for high-risk proliferative DR were 1. Optic disk neovascularization of ≥1/4 to 1/3 of the optic disk diameter, with or without preretinal hemorrhage or vitreous hemorrhage; 2. Preretinal hemorrhage or vitreous hemorrhage with optic disk neovascularization or retinal neovascularization of ≥1/4 to 1/3 of the optic disk diameter (11). Inclusion criteria were (1) Patients who were diagnosed with high-risk proliferative DR; (2) Type 2 diabetes patients with adequately controlled blood sugar, glycated hemoglobin (GHb) of less than 10%, blood pressure of less than 160/90 mm Hg (1kpa = 7.5 mm Hg); (3) Patients who did not receive prior fundus therapy such as retinal photocoagulation, anti-vascular endothelial growth factor intravitreal injections, or hormones. Exclusion criteria were: (1) Patients with type 1 diabetes; (2) Patients with poor imaging quality due to refractive interstitial opacity; (3) Patients with non-diabetic retinal vascular disease (these patients were excluded if fundus observation was affected by refractive interstitial opacity due to massive vitreous hemorrhage). All patients provided informed consent and were aware of the possible risks associated with the treatment (see Figure 1).

**Table 1:** General characteristics of patients.

Sex	
Male	28 (47.5%)
Female	31 (52.5%)
Age	61.84 ± 4.750
Laterality of the eye	
Right eye	29 (49.2%)
Left eye	30 (50.8%)
BCVA (LogMAR)	0.65 ± 0.18
CFT (μm)	456.53 ± 51.49
History of diabetes (years)	7.90 ± 1.41

### 2.2. Methods

Upon review of medical records, the data regarding age, sex, laterality of the eye. BCVA, central foveal thickness (CFT), retinal venous beading and retinal vein diameter were collected. The BCVA, CFT, retinal venous beading and retinal vein diameter at 6-, 12-, 18-, and 24 months post-treatment were compared with their pre-treatment measurements. All results were reviewed by the same senior ophthalmologist.

**Figure 2:**
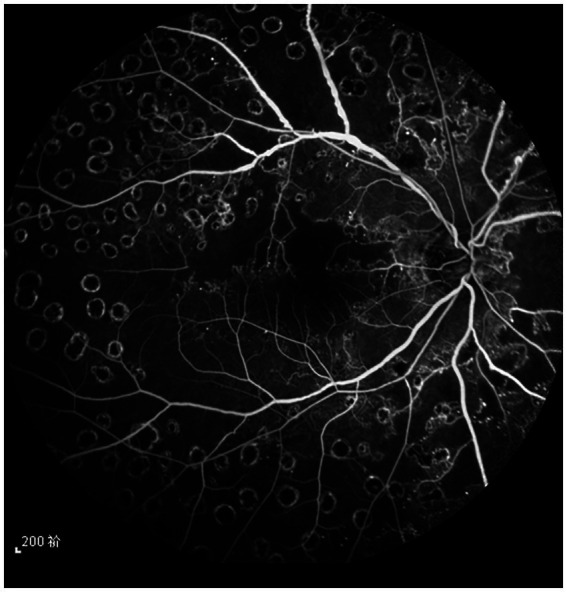
The fundus angiography image of the patient’s right eye 6 months after treatment, and the retinal laser spot is clear.

Intravitreal injection of aflibercept (2 mg) was administered to all patients monthly for the first 3 months adopting a 3 + PRN regimen. All injections were administered by the same senior physician. Eye drops of 0.5% levofloxacin were administered four times daily, starting 3 days before the surgery. Intravitreal injections of aflibercept and optical coherence tomography (HD-OCT) (Carl Zeiss AG) were performed by the same physician. Specifically, the eyes received aflibercept intravitreal injection + PRP at 0, 1, and 2 months. If NV (neovascular) persisted and/or if it recurred, combination therapy was administered for at least 4 weeks. For all study groups, the treatment standard was followed according to the ETDRS protocol for diabetic macular edema. Panretinal photocoagulation was performed 1 week after the first intravitreal injection (see Figure 2).

The affected eye was dilated with 1% tropicamide 15 min prior to examination. Fundus fluorescein angiography (FFA) was performed to obtain images of each patient.

The measurements of all venules passing through a zone with 1–1.5-disk diameters from the optic disc margin were taken from the macula-centered and optic disk-centered photographs. The calibers of the largest six veins were considered as the central retinal vein equivalent (CRVE) using the formula developed by Parr and Hubbard and revised by Knudtson (12). These equivalents were considered projected calibers for the central retinal veins. The intra-class reproducibility of retinal vascular measurements was excellent (the intra-class correlation coefficients for CRVEs all >0.98) in this study.

Fundus fluorescein angiography images were performed using a Heidelberg Spectralis HRA fundus camera and video angiography. The OCT images were acquired with the ZEISS Cirrus HD-OCT and measurements were taken using the built-in image processing software. The images with prominent retinal vein contours in the prevenous phase were used in fundus fluorescein angiography to avoid the interference of neovascular leakage with the measurements. All patients provided informed consent for the diagnostic and clinical procedures.

**Figure 3:**
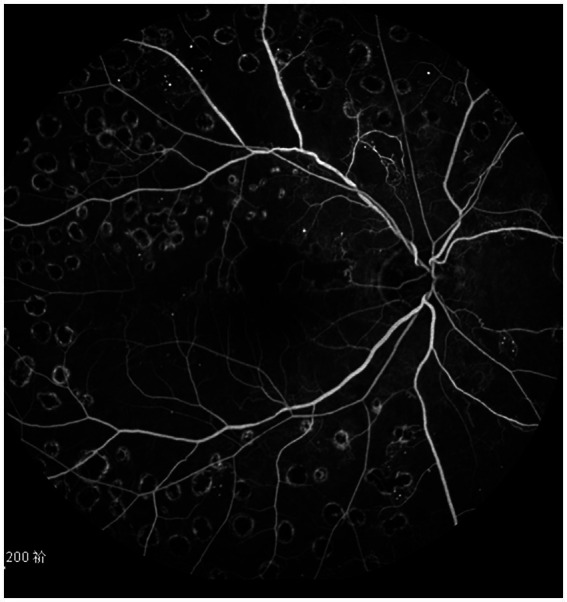
The fundus angiography image of the patient’s right eye 18 months after treatment, and the beading of the superior temporal branch retinal vein is significantly improved.

### 2.3. Panretinal photocoagulation

Wide-angle panretinal photocoagulation (PRP) was carried out. Briefly, a frequency-doubling 532 laser photocoagulator was used at a laser setting of 200 μm spot diameter, 200 ms pulse duration, and 230 mW power with a 165° retinoscope. The PRP protocol followed the guidelines formulated by the diabetic retinopathy photocoagulation group. The pupils were dilated enough before treatment. The laser started from the posterior pole with photocoagulation applied to the vicinity of the optic disc, 1PD nasally at the center of the macula, and beyond 2PD from the temporal side and anterior to the equator. The laser spots were aimed at 1 spot diameter apart. A total of 1,200–1,600 laser spots were delivered causing a level 3 burn and completed in four stages.

### 2.4. Statistical analysis

All statistical analyzes were performed using SPSS version 21.0 statistical software. Continuous variables were expressed as mean ± standard deviation, whereas categorical variables were expressed as percentages (%). The BCVA, CFT, and retinal vein diameter pre-treatment vs. post-treatment were compared using paired t-tests for the measurement data that conformed to normal distribution. The nonparametric test was used for the measurement data that did not conform to normal distribution. The ratios were tested using the Chi-Squared test. A value of p of less than 0.05 was considered statistically significant.

## 3. Results

The best corrected visual acuity (BCVA) at 6 months (0.49 ± 0.14 logMAR), 12 months (0.54 ± 0.15 logMAR), 18 months (0.48 ± 0.15 logMAR), and 24 months (0.51 ± 0.15 logMAR) post-treatment were superior to the pre-treatment measurement (0.65 ± 0.18 logMAR) (Table 2); the central foveal thickness (CFT) at 6 months (310.67 ± 52.53 μm), 12 months (295.98 ± 45.65 μm), 18 months (282.56 ± 43.57 μm) and 24 months (281.53 ± 51.16 μm) post-treatment was lower than the pre-treatment measurement (456.53 ± 51.49 μm) (Table 3); the retinal vein diameter at 12 months (310.13 ± 24.60 μm), 18 months (309.50 ± 31.58 μm), and 24 months (317.00 ± 27.54 μm) post-treatment was lower than the pre-treatment measurement (361.81 ± 30.26 μm) (Table 4). The retinal venous beading changed significantly at 18 months post-treatment, with decreased beading (see Figure 3).

**Table 2:** Comparison of the BCVA (LogMAR) pre-treatment vs. post-treatment.

	Pre-treatment	6 months post-treatment	12 months post-treatment	18 months post-treatment	24 months post-treatment
*n* = 59	0.65 ± 0.18	0.49 ± 0.14	0.54 ± 0.15	0.48 ± 0.15	0.51 ± 0.15
*t*		5.3909	3.5093	5.5043	4.4043
*p*		0.0000	0.0006	0.0000	0.0000

**Table 3:** Comparison of the CFT (μm) pre-treatment vs. post-treatment.

	Pre-treatment	6 months post-treatment	12 months post-treatment	18 months post-treatment	24 months post-treatment
*n* = 59	456.53 ± 51.49	310.67 ± 52.53	295.98 ± 45.65	282.56 ± 43.57	281.53 ± 51.16
*t*		15.2314	17.9211	19.8110	18.5187
*p*		0.0000	0.0000	0.0000	0.0000

**Table 4:** Comparison of the retinal vein diameter(μm) pre-treatment vs. post-treatment.

	Pre-treatment	6 months post-treatment	12 months post-treatment	18 months post-treatment	24 months post-treatment
*n* = 59	361.81 ± 30.26	352.98 ± 24.59	310.13 ± 24.60	309.50 ± 31.58	317.00 ± 27.54
*t*		1.7402	10.1792	9.1875	8.4121
*p*		0.0845	0.0000	0.0000	0.0000

## 4. Discussion

Diabetic retinopathy occurs in two forms, non-proliferative diabetic retinopathy (NPDR) and proliferative diabetic retinopathy (PDR). This study elucidated the changes in retinal veins (venous beading) and the changes in retinal vein calibers in high-risk PDR patients. Fundoscopy, OCT, and fundus fluorescein angiography (FFA) techniques were used for observing the changes. Filho et al. compared intravitreal 0.5 mg ranibizumab withPRP versus PRP alone for the treatment of high-risk PDR in 40 patients. They found significant reduction in fluorescein angiography leakage in both groups through week 48, but the reduction was significantly greater in the combination group, along with significant improvement in visual acuity and central retinal thickness (13), which is the same as our results in terms of visual acuity and central foveal thickness.

CLARITY was a multi-center phase 2b, single-blind, randomized, noninferiority trial that compared aflibercept to PRP. At 52 weeks, aflibercept was not only noninferior to PRP but also superior to PRP in terms of visual change. New-onset centers involved DME, vitreous hemorrhage, need for vitrectomy, and visual loss were more likely to occur in eyes treated with PRP than with aflibercept (14). The special feature of our study is to observe the changes of retinal vein diameter and vein beading during treatment.

Baseline Retinopathy and Clinical Features Predict Progression of Diabetic Retinopathy showed that baseline signs and initial DR were prognostic from report 3 of the 2017 United Kingdom Diabetic Retinopathy Electronic Medical Record Users Group. It concluded that IRMA increases the risk of PDR whereas 4Q DBH increases the risk of VH. Venous beading was not a critical variable as the other two features in predicting PDR or VH (15) In this study, a significant remission of retinal venous beading and a reduction in retinal venous diameter by fundus photography and fundus fluorescein angiography in patients with PDR following intravitreal injections of anti-VEGF agents were noticed.

As the DR progressed, the death of pericytes and the thickening of the basement membrane resulted in impaired perfusion and retinal ischemia. The increased ischemia led to the formation of VB (16) A previous domestic study showed that VB was the chronic reactive expansion of the retinal vein in response to retinal ischemia or other abnormal stimuli (17). The ancillary studies of CLARITY revealed that aflibercept reduced retinal hemorrhages and intravitreal microvascular abnormalities but not venous beading at week 52, suggesting that VEGF would not have been involved in the pathophysiology of vein changes, or these anatomical changes may not have been improved in a relatively short period of 1 year (18).

The retinal blood flow and hydrostatic pressure in the retinal vessels were increased in diabetic retinopathy (19). The increased hydrostatic pressure in the retinal vessels may have been responsible for the small retinal vessel expansion. In this study, significant changes in the retinal venous beading were noted in PDR patients at 18 months, which may have been due to the anatomical changes in the retinal vein which may have required a sufficiently long time for the vein remodeling. On the other hand, retinal veins had no venous valves and the venous beading may have improved after remission of retinal ischemia and reduction of hydrostatic pressure in the retinal vessels.

A 12-month prospective clinical trial found that the calibers of both retinal arterioles and venules were reduced by the intravitreal anti-vascular endothelial growth factor (VEGF) treatment in DME, and the eyes that did not even receive PRN aflibercept after the loading phase had sustainable venous constriction at 12 months. The favorable effect of anti-VEGF therapy on retinal thickness in DME treatment might have been at least in part attributable to the reduction of pathologically increased vessel calibers to normal levels and a subsequent decrease in hydrostatic pressure (20). Abnormal diameter in diabetes due to the changes in perfusion pressure might have been attributed to a lack of vascular tone and changes in the vessel walls. Additionally, endothelial dysfunction may have led to impaired endothelial vasodilatation and an imbalance of retinal vessel diameter regulation in diabetic patients (21). Recent evidence also found that retinal glial cells were capable of sensing the reduction in perfusion pressure and contributed to the maintenance of vessel diameters. The glial cells are affected early in the diabetic retina and continue to degenerate along with retinal ganglion cells.

We speculate that the inflammatory response is also an important part of the mechanism of the effect of intravitreal injection of aflibercept on the retinal vein diameter and venous beading.aflibercept, by binding also to PlGF, could exert an anti-inflammatory action in the diabetic retina (22).

This study has the following limitations. First, an untreated control group was not included in the analysis for ethical reasons. Second, the effect of aging on retinal vascular remodeling was not ruled out (an elderly population was elucidated), although the blood pressure parameters were well controlled.

Furthermore, wider retinal vein calibre was considered an independent risk factor for the subsequent occurrence and development of DR (23).

In high-risk PDR patients who received anti-VEGF treatments, statistically significant differences in the BCVA and CFT were noticed at 6 months, 12 months, 18 months, and 24 months post-operation compared with the pre-operative measurements (*p* < 0.05). Statistically, significant differences in the retinal vessel diameter were observed at 12 months, 18 months, and 24 months post-operation compared with the pre-operative measurements (p < 0.05). The retinal venous beading improved significantly at 18 months. The anatomical changes of the retinal vein required a sufficiently long time for vein remodeling. Studies with a longer follow-up are warranted for further investigation in the future.

## 5. Conclusion

Aflibercept intravitreal injection and panretinal photocoagulation may reverse the retinal vein diameter and venous beading in high-risk proliferative diabetic retinopathy.

## Data availability statement

The original contributions presented in the study are included in the article/supplementary material, further inquiries can be directed to the corresponding authors.

## Ethics statement

This study was conducted after approval by the Ethics Committee of Qilu Hospital of Shandong University. The patients/participants provided their written informed consent to participate in this study.

## Author contributions

HZ, HW, and YT carried out the conception and design of the research and drafted the manuscript. HZ and SL participated in obtaining funding. JW and YT participated in the acquisition of data. YB and XZ carried out the analysis and interpretation of data. HW and YT participated in the design of the study and performed the statistical analysis. All authors contributed to the article and approved the submitted version.

## Conflict of interest

The authors declare that the research was conducted in the absence of any commercial or financial relationships that could be construed as a potential conflict of interest.

## Publisher’s note

All claims expressed in this article are solely those of the authors and do not necessarily represent those of their affiliated organizations, or those of the publisher, the editors and the reviewers. Any product that may be evaluated in this article, or claim that may be made by its manufacturer, is not guaranteed or endorsed by the publisher.
